# Imported case of Dengue virus 3 genotype I in Rio de Janeiro state, Brazil

**DOI:** 10.1590/0074-02760180036

**Published:** 2018-06-25

**Authors:** Marcos César Lima de Mendonça, Maria Angelica Mares-Guia, Cintia Damasceno dos Santos Rodrigues, Carolina Cardoso dos Santos, Flavia Lowen Levy Chalhoub, Eliane Saraiva Machado Araújo, Alexandre Otávio Chieppe, Rita Maria Ribeiro Nogueira, Ana Maria Bispo de Filippis

**Affiliations:** 1 Fundação Oswaldo Cruz-Fiocruz, Instituto Oswaldo Cruz, Laboratório de Flavivírus, Rio de Janeiro, RJ, Brasil; 2 Secretaria de Estado de Saúde do Rio de Janeiro, Rio de Janeiro, RJ, Brasil

**Keywords:** dengue, sequencing, genotyping

## Abstract

The dengue virus (DENV), of the genus *Flavivirus* (*Flaviviridae*), has four antigenically distinct serotypes, of which DENV-3 is classified into five genotypes. Here, we describe the detection of DENV-3 genotype I in sera of a Brazilian patient travelling from Singapore to Rio de Janeiro, Brazil, by using multiplex real-time RT-PCR, DNA sequencing of the whole envelope protein gene, and phylogenetic analysis. The virus shares ancestry with those identified in Bali, Indonesia, in 2015. It is possible that arboviruses such as Chikungunya ECSA genotype, DENV-4 genotype I, and Zika were introduced in Brazil from other continents during the multiple international events hosted by the country over the last four years, including World Youth Day, the Soccer World Cup, and the Summer Olympics.

Dengue virus (DENV) is considered the most important arbovirus worldwide, and approximately two-fifths of the world's population live in areas endemic for dengue (Guo et al. 2017). DENV is classified into family *Flaviviridae*, genus *Flavivirus*, and it comprises four antigenically distinct serotypes: Dengue virus 1 (DENV1), Dengue virus 2 (DENV2), Dengue virus 3 (DENV3), and Dengue virus 4 (DENV4). DENV is transmitted to humans by mosquitoes belonging to genus *Aedes*, mainly *Aedes aegypti*, which are prevalent in tropical and subtropical areas (Whitehead et al. 1971, Lanciotti et al. 1994).

Phylogenetic studies focusing on the envelope protein proposed the further classification of DENV-3 into four genotypes (genotypes I, II, III, and IV). The first discovered genotype of DENV-3, Genotype IV, was first identified in the Caribbean and Puerto Rico in 1963 (Lanciotti et al. 1994) and was, at one point, the only one circulating in different countries in the Americas; however, it may currently be extinct. Viruses classified as Genotype I were found in Colombia in 2003 (Usme-Ciro et al. 2008). In Brazil, similar viruses were detected in 2003, but the authors proposed a new genotype (genotype V) for the correct classification of these viruses owing to their topology in the phylogenetic tree (Aquino et al. 2009). According to this classification, Genotype V viruses were present in Brazil and Colombia between 2003 and 2006. They were closely related to viruses from Asian samples isolated in approximately 1956, and until that date, there was no record of their presence in nature (Aquino et al. 2009). Genotype III was introduced in Latin America in the early 1990s (Araújo et al. 2009) and in Brazil in 2001 (Miagostovich et al. 2002), and it has since become the predominant genotype. No evidence exists supporting the current circulation of genotype II in the Americas.

This work was performed in accordance with ethical committee CAAE: 57221416.0.1001.5248. Between May 2015 and August 2017, 733 acute sera samples were collected as part of epidemiologic surveillance and tested for dengue by real-time RT-PCR using the protocol described by Santiago et al. (2013). The protocol is a multiplex real-time PCR that differentiates the four serotypes of Dengue. Each pair of primer and Taqman probe was designed for different regions of the genome of the virus as per the virus searched. For DENV3, the chosen region is within the coding region of prM, and only one sample was positive for DENV3, presenting a Ct value of 23. All samples presented negative results when tested for DENV 1, 2 and 4. The DENV3-positive sample was obtained from a 36-year-old Brazilian who lives in Singapore. During his 30-day vacation, he toured Indonesia and other Asian countries. The last country visited was Brazil. The patient arrived in Brazil, four days after his return presenting symptoms such as fever, prostration and retro-orbital pain, without complications. The patient sought medical assistance and stayed at home, in an urban area of Rio de Janeiro, for two weeks until the symptoms disappeared.

The Qiagen Onestep RT-PCR Kit was used to generate a 1795-bp fragment that contained the entire coding region for the envelope protein. Ten primers were used for Sanger sequencing reactions (Data not shown). The MEGA 7 software package was used to choose the best model for nucleotide substitution and to construct the phylogenetic trees.

The tree shown in Figure (A) was constructed using 2,040 sequences, obtained by Blast (Available from: https://blast.ncbi.nlm.nih.gov/Blast.cgi), of the envelope protein coding region from all five genotypes. In Figure (A), the sequenced DENV3+ virus, identified as Brazil-RJ/2017/LABFLA (accession number MG812331), belongs to genotype I among samples from Indonesia and Singapore. This virus is in a cluster with four other samples that were described in a study with samples from Bali, Indonesia, collected in 2015 (Megawati et al. 2017) Therefore, viruses from Bali, Indonesia, and those from the DENV3+ patient (Brazil-RJ/2017/LABFLA) in this study have common ancestry. When the sequence of this patient's virus was compared with KY006150.1, i.e., the sequence with the shortest distance, we verified four nucleotide changes, but there was no amino acid change for the E protein region.

**Figure:**
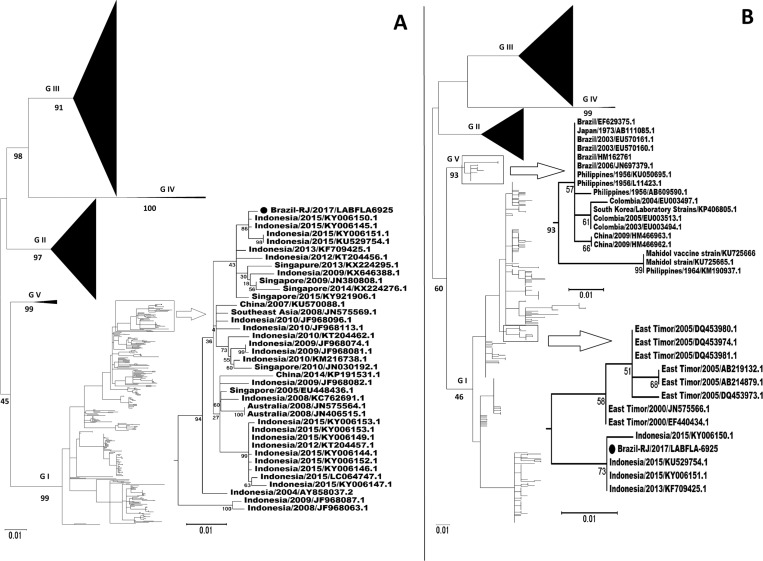
Phylogenetic analyses based on complete (A) and partial (B - 224-bp 3- terminal) protein E coding region sequences were performed in MEGA 7. Closed black circle represents the strain identified in the patient (Brasil-RJ/2017/LABFLA). The trees were inferred with 1000 bootstrap replicates, using maximum likelihood algorithm based on GTR model with Gamma distribution and invariant site correction.

A second tree (B) was constructed because there were no complete envelope protein sequence deposits from the Colombian samples classified as genotype I (reclassified as genotype V). The second tree was assembled with 224 3'-terminal nucleotides of the envelope protein coding region. The phylogenetic tree clearly indicates the genotype IV virus's introduction into Colombia has no correlation with the virus sample found in Rio de Janeiro. The DENV3 virus identified in this study was similar to the genotypes found in Bali and indicates that the patient could have acquired the infection in one of the countries that he visited during his trip in Southeast Asia.

Again, our results show that Brazil has become an important gateway to arboviruses from other continents, as seen with Zika, Yellow Fever, Chikungunya genotype ECSA, DENV-4 genotype I and, potentially, DENV-3 genotype I. The identification of DENV3 genotype I in this patient is an indication that the health authorities should be aware of this new strain introduction and intensify their DENV serotype and genotype surveillance.
